# A Modern Idea of Gout

**Published:** 1919-05-03

**Authors:** 


### A MODERN IDEA OF GOUT.

ONE is nowadays so used to repeated summaries of our position in relation to acute infections, necessary owing to the extraordinary speed with which modern experimental work has lately progressed, that there is some risk of forgetting the more homely pathological states which in the past filled a large part of the list of family ailments and demanded then, as now, much attention from the home practitioner. Coming at this time, a small volume on Gout, by Dr. Berkart, and published by the Oxford University Press, gives not only a needed reminder of our lack of exact knowledge, but also a useful synopsis of available treatment for the conditions.

Several of his points are well worthy of attention, not for the novelty of their character, but for the attack which is made on those fixed ideas which still surround gout and many similar subacute states. A diagnosis of gout must not necessarily be the signal for immediate change in the patient's mode of life unless this has some obvious faults. The question of feeding is chiefly important in that thereby can the patient's health and bodily tone be improved, his metabolism quickened, and lethargy or obesity reduced, but each case involves its own problems and must be judged by its own standards. There is the too well-known experience that vegetarianism will suit not more than a limited percentage of people and benefit still fewer. Similarly the non-gouty diet shows extreme variations in its effects.

In the instance of alcoholic drinks, nothing is as yet ascertained whether and in what manner they affect the formation and excretion of uric acid. The effect of taking of alcohol appears to show an exact parallel to the uncertain effect of the meat-free diet. In some parts of Germany gout is unknown. Beer would be injurious to those of the gouty who are gross or inclined to obesity, while it is harmless or even beneficial to the emaciated, whose body needs all possible nourishment and strength.

And here one may recall the fact that gout is always accepted as a disease of the robust and overfed, but reflection will prove that in hospital practice any number of instances are seen in the older inmates, where an extremely different picture is displayed. It would almost seem that gout is a disease of low, stagnated vitality, that a case may best be treated by momentarily forgetting the local condition and unravelling the mystery of the patient's imperfect physical state. Somewhere his machinery is inefficient, and where, apart from actual senility, can the weakness be found? Improve, aid, or repair this and the power of gout, if real gout it be, will be largely neutralised.

Then our usual palliative, internal and external methods of supposed uric acid solution, may be marshalled. Dr. Berkart, in his summary of these, does justice to each and allows it whatever credit it can rightly claim, but, nevertheless, the reading of this booklet suggests that gout should be viewed as a local pathological condition aggravated, if not caused, by stagnation of the human machine, and hence to be attacked along lines of rational hygiene and health rather than by the physic of old times.

